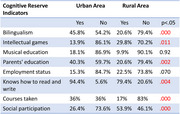# Cognitive Reserve Indicators Among Older Adults Living in Urban and Rural Areas of Jalisco, Mexico

**DOI:** 10.1002/alz70861_108264

**Published:** 2025-12-23

**Authors:** Melina Rodríguez Díaz, Neyda Ma. Mendoza Ruvalcaba, Alessandra C Díaz

**Affiliations:** ^1^ Universidad de Guadalajara, Guadalajara, JA Mexico; ^2^ Universidad de Guadalajara CUTONALA, Tonala, JA Mexico

## Abstract

**Background:**

Reserve has evolved in recent years by incorporating terms such as brain reserve, cognitive reserve, brain compensation, and brain maintenance, which has complicated its study and the establishment of a consensual definition of its indicators. Access to these indicators depends on the resources available to the aging population.

**Method:**

This is a cross‐sectional study. The sample was drawn from the project *Cognitive Functioning of Older Adults in the Urban Area of Guadalajara* (Project‐Conacyt‐256589), with a total of *n = 72* older adults (𝑥̄ = 75.47, SD = 6.66 years, 58% women), and from the rural area, with a total of *n = 141* older adults (𝑥̄ = 71.87, SD = 8.66, 73.5% women).

**Result:**

Findings show that the cognitive reserve indicators that significantly favored (p < .05) older adults living in the urban area, compared to those living in the rural area, were education, parents’ education, training courses, social participation, and bilingualism. Conversely, rural participants scored significantly higher in intellectual games. Finally, although the urban group had slightly higher mean scores in global cognitive functioning, the difference was not statistically significant.

**Conclusion:**

This study reveals differences in cognitive reserve indicators among older adults living in populations with either favorable or limiting characteristics that influence optimal functional and cognitive maintenance. These findings highlight the importance of ensuring access to educational resources, social participation opportunities, and overall health services for individuals living and growing up in rural areas of Mexico to promote better cognitive aging outcomes.